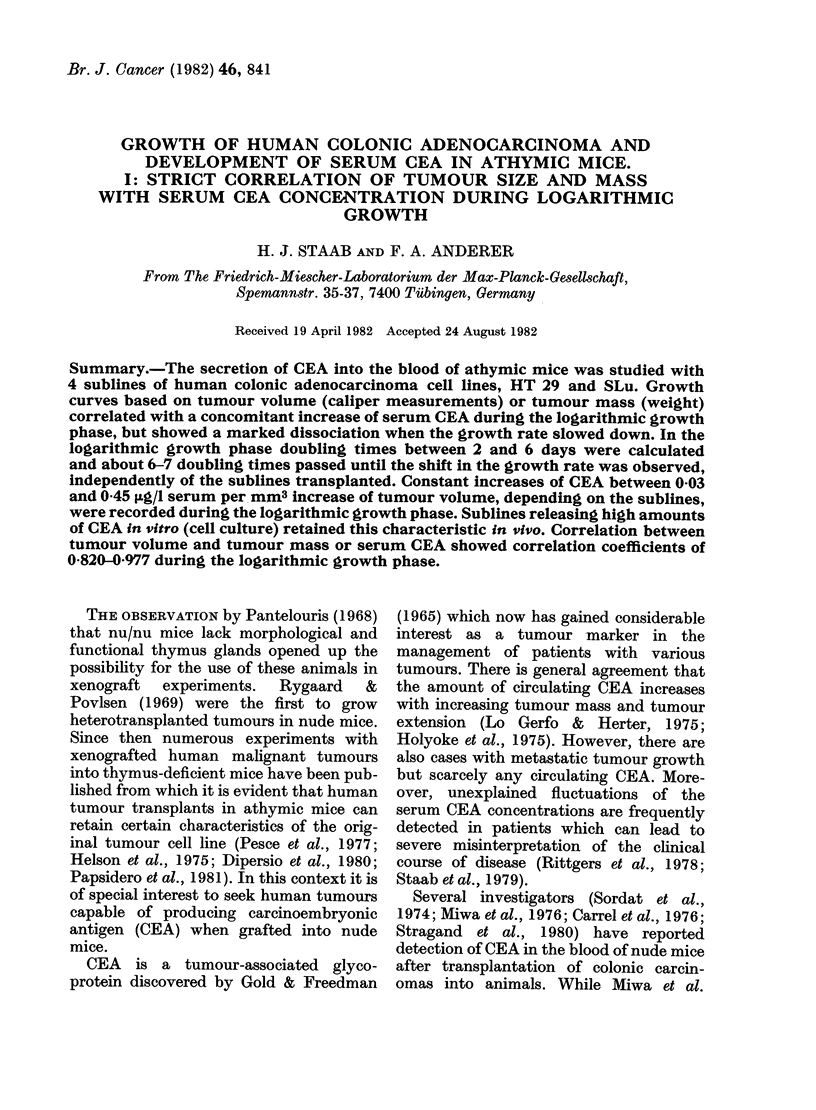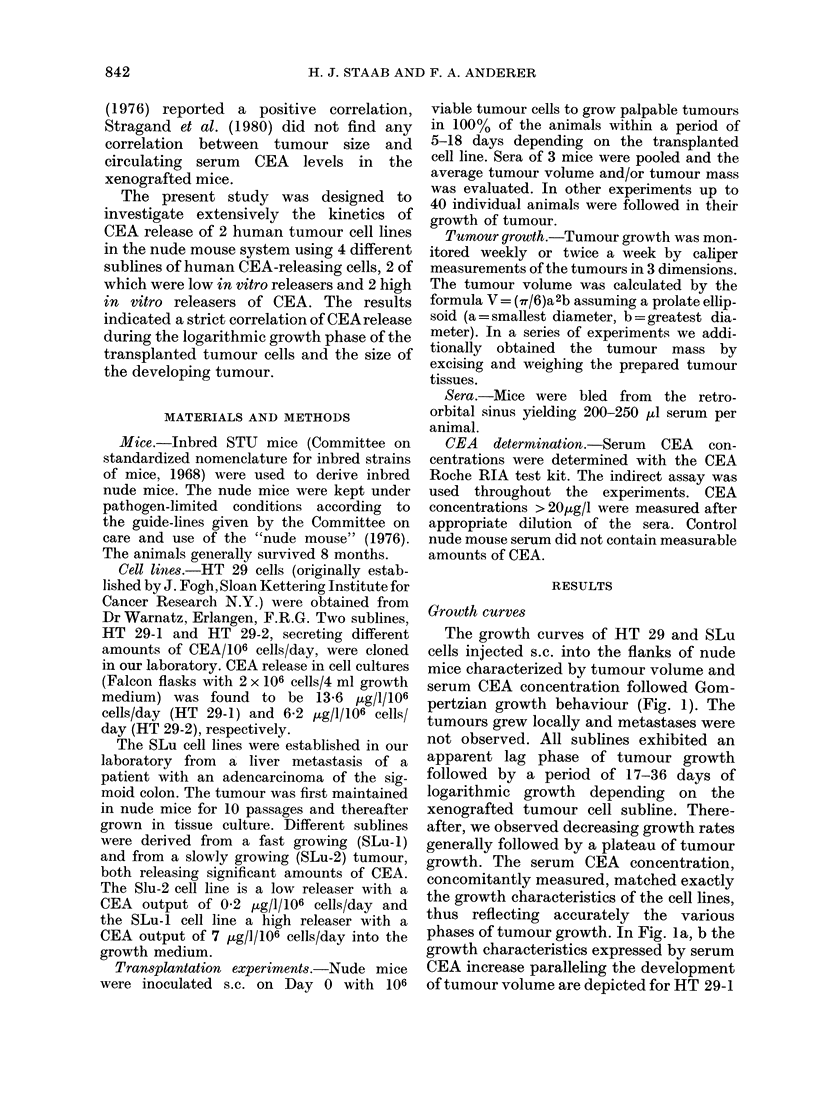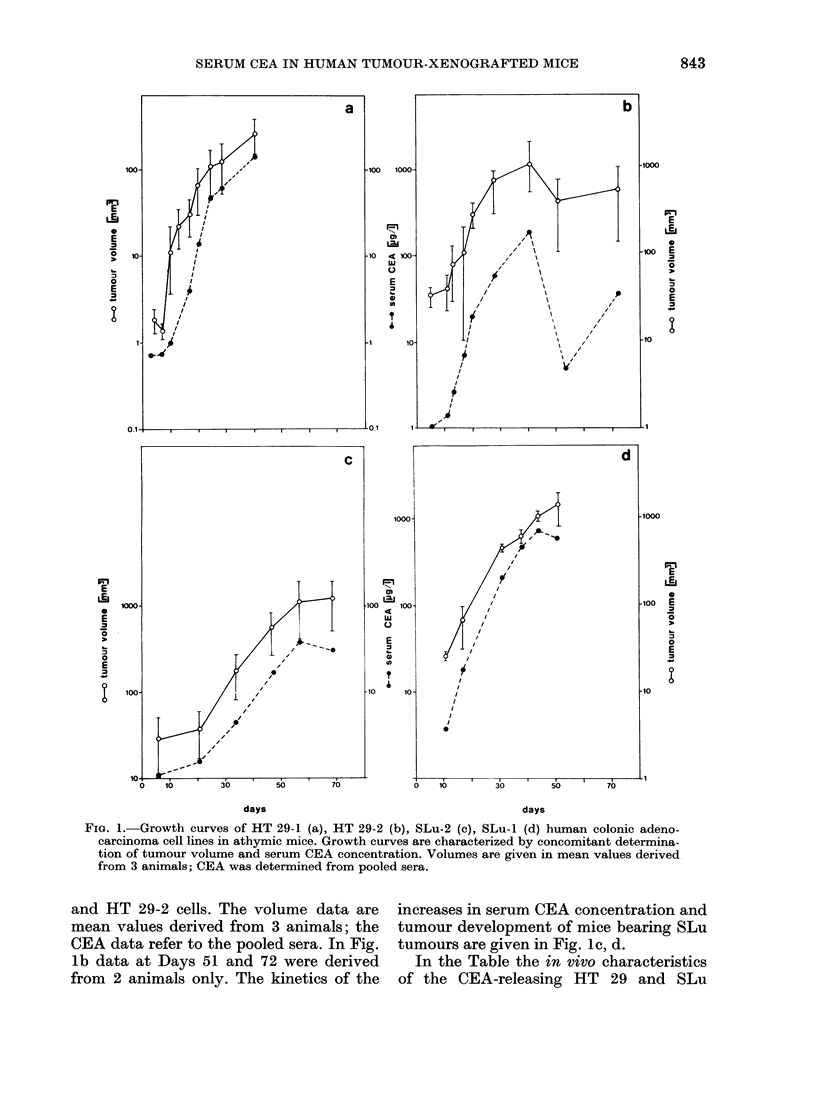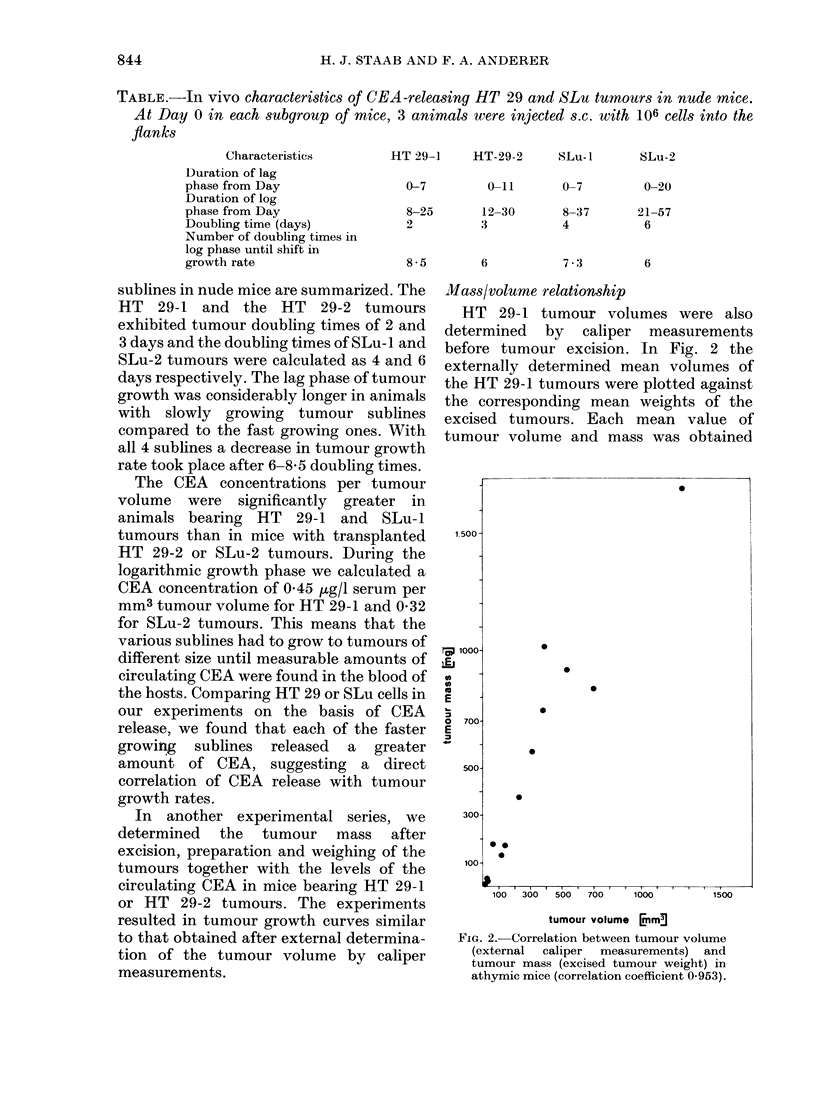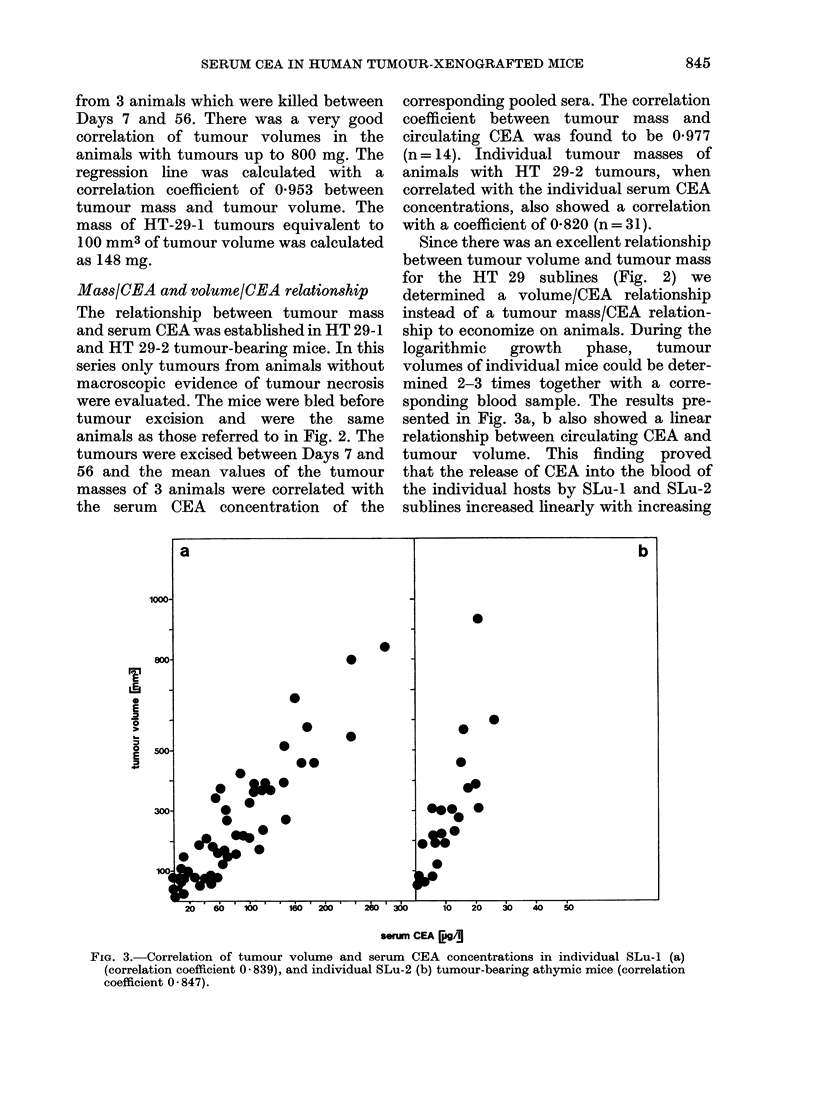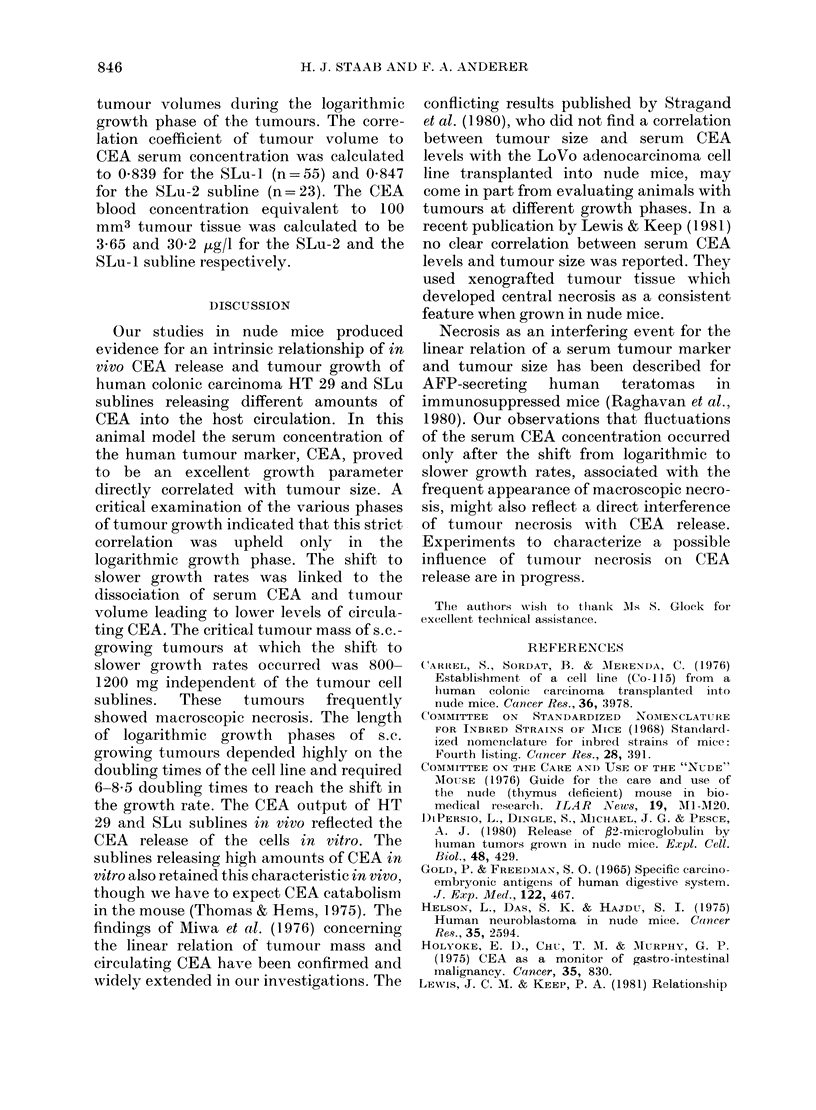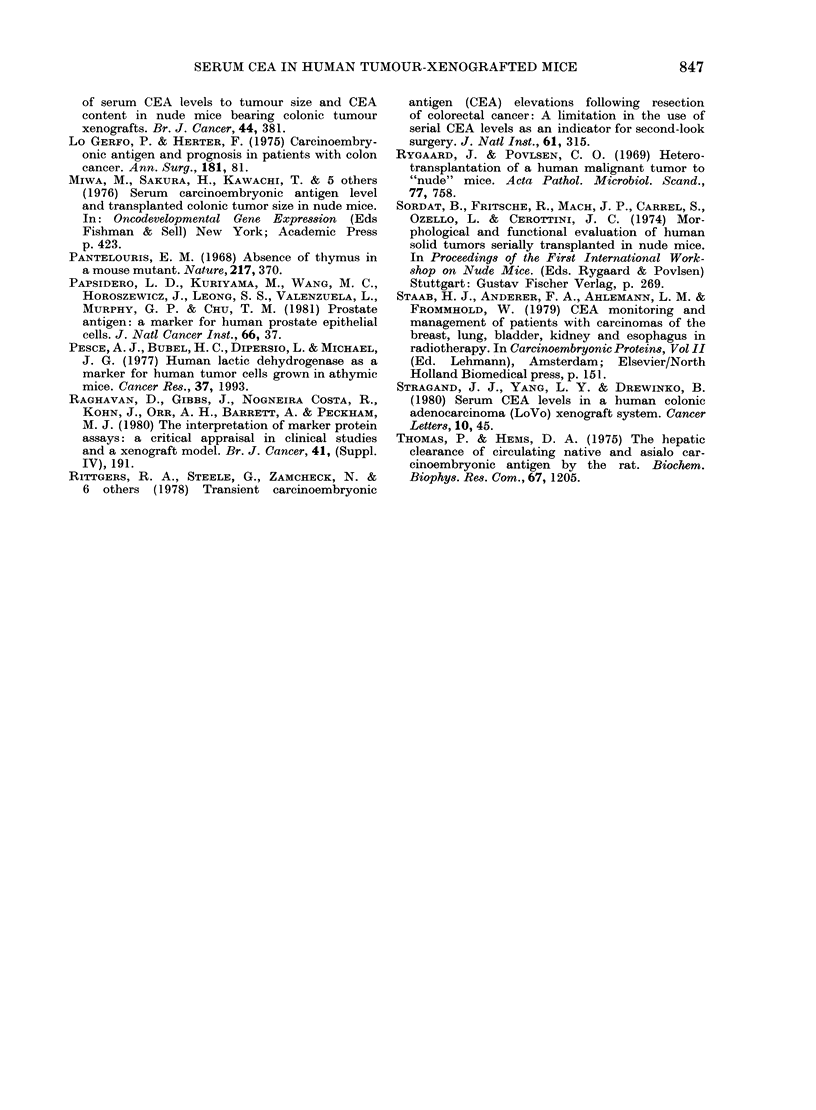# Growth of human colonic adenocarcinoma and development of serum CEA in in athymic mice. I: Strict correlation of tumour size and mass with serum CEA concentration during logarithmic growth.

**DOI:** 10.1038/bjc.1982.293

**Published:** 1982-12

**Authors:** H. J. Staab, F. A. Anderer

## Abstract

The secretion of CEA into the blood of athymic mice was studied with 4 sublines of human colonic adenocarcinoma cell lines, HT 29 and SLu. Growth curves based on tumour volume (caliper measurements) or tumour mass (weight) correlated with a concomitant increase of serum CEA during the logarithmic growth phase, but showed a marked dissociation when the growth rate slowed down. In the logarithmic growth phase doubling times between 2 and 6 days were calculated and about 6-7 doubling times passed until the shift in the growth rate was observed, independently of the sublines transplanted. Constant increases of CEA between 0.03 and 0.45 microgram/l serum per mm3 increase of tumour volume, depending on the sublines, were recorded during the logarithmic growth phase. Sublines releasing high amounts of CEA in vitro (cell culture) retained this characteristic in vivo. Correlation between tumour volume and tumour mass or serum CEA showed correlation coefficients of 0.820-0.977 during the logarithmic growth phase.


*Br. J. Cancer* (1982) **46**, 841

### GROWTH OF HUMAN COLONIC ADENOCARCINOMA AND DEVELOPMENT OF SERUM CEA IN ATHYMIC MICE. I: STRICT CORRELATION OF TUMOUR SIZE AND MASS WITH SERUM CEA CONCENTRATION DURING LOGARITHMIC GROWTH

H. J. STAAB AND F. A. ANDERER

*From The Friedrich-Miescher-Laboratorium der Max-Planck-Gesellschaft, Spemannstr.* 35-37, 7400 *Tübingen, Germany*

Received 19 April 1982 Accepted 24 August 1982

**Summary.—The secretion of CEA into the blood of athymic mice was studied with 4 sublines of human colonic adenocarcinoma cell lines, HT 29 and SLu. Growth curves based on tumour volume (caliper measurements) or tumour mass (weight) correlated with a concomitant increase of serum CEA during the logarithmic growth phase, but showed a marked dissociation when the growth rate slowed down. In the logarithmic growth phase doubling times between 2 and 6 days were calculated and about 6–7 doubling times passed until the shift in the growth rate was observed, independently of the sublines transplanted. Constant increases of CEA between 0·03 and 0·45 μg/l serum per mm³ increase of tumour volume, depending on the sublines, were recorded during the logarithmic growth phase. Sublines releasing high amounts of CEA *in vitro* (cell culture) retained this characteristic *in vivo*. Correlation between tumour volume and tumour mass or serum CEA showed correlation coefficients of 0·820–0·977 during the logarithmic growth phase.**

THE OBSERVATION by Pantelouris (1968) that nu/nu mice lack morphological and functional thymus glands opened up the possibility for the use of these animals in xenograft experiments. Rygaard & Povlsen (1969) were the first to grow heterotransplanted tumours in nude mice. Since then numerous experiments with xenografted human malignant tumours into thymus-deficient mice have been published from which it is evident that human tumour transplants in athymic mice can retain certain characteristics of the original tumour cell line (Pesce *et al.*, 1977; Helson *et al.*, 1975; Dipersio *et al.*, 1980; Papsidero *et al.*, 1981). In this context it is of special interest to seek human tumours capable of producing carcinoembryonic antigen (CEA) when grafted into nude mice.

CEA is a tumour-associated glycoprotein discovered by Gold & Freedman (1965) which now has gained considerable interest as a tumour marker in the management of patients with various tumours. There is general agreement that the amount of circulating CEA increases with increasing tumour mass and tumour extension (Lo Gerfo & Herter, 1975; Holyoke *et al.*, 1975). However, there are also cases with metastatic tumour growth but scarcely any circulating CEA. Moreover, unexplained fluctuations of the serum CEA concentrations are frequently detected in patients which can lead to severe misinterpretation of the clinical course of disease (Rittgers *et al.*, 1978; Staab *et al.*, 1979).

Several investigators (Sordat *et al.*, 1974; Miwa *et al.*, 1976; Carrel *et al.*, 1976; Stragand *et al.*, 1980) have reported detection of CEA in the blood of nude mice after transplantation of colonic carcinomas into animals. While Miwa *et al.*

(1976) reported a positive correlation, Stragand *et al.* (1980) did not find any correlation between tumour size and circulating serum CEA levels in the xenografted mice.

The present study was designed to investigate extensively the kinetics of CEA release of 2 human tumour cell lines in the nude mouse system using 4 different sublines of human CEA-releasing cells, 2 of which were low *in vitro* releasers and 2 high *in vitro* releasers of CEA. The results indicated a strict correlation of CEA release during the logarithmic growth phase of the transplanted tumour cells and the size of the developing tumour.

### MATERIALS AND METHODS

*Mice.*—Inbred STU mice (Committee on standardized nomenclature for inbred strains of mice, 1968) were used to derive inbred nude mice. The nude mice were kept under pathogen-limited conditions according to the guide-lines given by the Committee on care and use of the "nude mouse" (1976). The animals generally survived 8 months.

*Cell lines.*—HT 29 cells (originally established by J. Fogh, Sloan Kettering Institute for Cancer Research N.Y.) were obtained from Dr Warnatz, Erlangen, F.R.G. Two sublines, HT 29-1 and HT 29-2, secreting different amounts of CEA/$10^6$ cells/day, were cloned in our laboratory. CEA release in cell cultures (Falcon flasks with $2 \times 10^6$ cells/4 ml growth medium) was found to be 13·6 $\mu$g/l/$10^6$ cells/day (HT 29-1) and 6·2 $\mu$g/l/$10^6$ cells/day (HT 29-2), respectively.

The SLu cell lines were established in our laboratory from a liver metastasis of a patient with an adencarcinoma of the sigmoid colon. The tumour was first maintained in nude mice for 10 passages and thereafter grown in tissue culture. Different sublines were derived from a fast growing (SLu-1) and from a slowly growing (SLu-2) tumour, both releasing significant amounts of CEA. The Slu-2 cell line is a low releaser with a CEA output of 0·2 $\mu$g/l/$10^6$ cells/day and the SLu-1 cell line a high releaser with a CEA output of 7 $\mu$g/l/$10^6$ cells/day into the growth medium.

*Transplantation experiments.*—Nude mice were inoculated s.c. on Day 0 with $10^6$ viable tumour cells to grow palpable tumours in 100% of the animals within a period of 5–18 days depending on the transplanted cell line. Sera of 3 mice were pooled and the average tumour volume and/or tumour mass was evaluated. In other experiments up to 40 individual animals were followed in their growth of tumour.

*Tumour growth.*—Tumour growth was monitored weekly or twice a week by caliper measurements of the tumours in 3 dimensions. The tumour volume was calculated by the formula $V = (\pi/6)a^2b$ assuming a prolate ellipsoid (a = smallest diameter, b = greatest diameter). In a series of experiments we additionally obtained the tumour mass by excising and weighing the prepared tumour tissues.

*Sera.*—Mice were bled from the retro-orbital sinus yielding 200–250 $\mu$l serum per animal.

*CEA determination.*—Serum CEA concentrations were determined with the CEA Roche RIA test kit. The indirect assay was used throughout the experiments. CEA concentrations $> 20 \mu$g/l were measured after appropriate dilution of the sera. Control nude mouse serum did not contain measurable amounts of CEA.

### RESULTS

### *Growth curves*

The growth curves of HT 29 and SLu cells injected s.c. into the flanks of nude mice characterized by tumour volume and serum CEA concentration followed Gompertzian growth behaviour (Fig. 1). The tumours grew locally and metastases were not observed. All sublines exhibited an apparent lag phase of tumour growth followed by a period of 17–36 days of logarithmic growth depending on the xenografted tumour cell subline. Thereafter, we observed decreasing growth rates generally followed by a plateau of tumour growth. The serum CEA concentration, concomitantly measured, matched exactly the growth characteristics of the cell lines, thus reflecting accurately the various phases of tumour growth. In Fig. 1a, b the growth characteristics expressed by serum CEA increase paralleling the development of tumour volume are depicted for HT 29-1

FIG. 1.—Growth curves of HT 29-1 (a), HT 29-2 (b), SLu-2 (c), SLu-1 (d) human colonic adenocarcinoma cell lines in athymic mice. Growth curves are characterized by concomitant determination of tumour volume and serum CEA concentration. Volumes are given in mean values derived from 3 animals; CEA was determined from pooled sera.

and HT 29-2 cells. The volume data are mean values derived from 3 animals; the CEA data refer to the pooled sera. In Fig. 1b data at Days 51 and 72 were derived from 2 animals only. The kinetics of the increases in serum CEA concentration and tumour development of mice bearing SLu tumours are given in Fig. 1c, d.

In the Table the *in vivo* characteristics of the CEA-releasing HT 29 and SLu

TABLE.—In vivo *characteristics of CEA-releasing HT 29 and SLu tumours in nude mice. At Day* 0 *in each subgroup of mice,* 3 *animals were injected s.c. with* $10^6$ *cells into the flanks*

| Characteristics | HT 29-1 | HT-29-2 | SLu-1 | SLu-2 |
|---|---|---|---|---|
| Duration of lag phase from Day | 0–7 | 0–11 | 0–7 | 0–20 |
| Duration of log phase from Day | 8–25 | 12–30 | 8–37 | 21–57 |
| Doubling time (days) | 2 | 3 | 4 | 6 |
| Number of doubling times in log phase until shift in growth rate | 8·5 | 6 | 7·3 | 6 |

sublines in nude mice are summarized. The HT 29-1 and the HT 29-2 tumours exhibited tumour doubling times of 2 and 3 days and the doubling times of SLu-1 and SLu-2 tumours were calculated as 4 and 6 days respectively. The lag phase of tumour growth was considerably longer in animals with slowly growing tumour sublines compared to the fast growing ones. With all 4 sublines a decrease in tumour growth rate took place after 6–8·5 doubling times.

The CEA concentrations per tumour volume were significantly greater in animals bearing HT 29-1 and SLu-1 tumours than in mice with transplanted HT 29-2 or SLu-2 tumours. During the logarithmic growth phase we calculated a CEA concentration of 0·45 μg/l serum per $mm^3$ tumour volume for HT 29-1 and 0·32 for SLu-2 tumours. This means that the various sublines had to grow to tumours of different size until measurable amounts of circulating CEA were found in the blood of the hosts. Comparing HT 29 or SLu cells in our experiments on the basis of CEA release, we found that each of the faster growing sublines released a greater amount of CEA, suggesting a direct correlation of CEA release with tumour growth rates.

In another experimental series, we determined the tumour mass after excision, preparation and weighing of the tumours together with the levels of the circulating CEA in mice bearing HT 29-1 or HT 29-2 tumours. The experiments resulted in tumour growth curves similar to that obtained after external determination of the tumour volume by caliper measurements.

*Mass/volume relationship*

HT 29-1 tumour volumes were also determined by caliper measurements before tumour excision. In Fig. 2 the externally determined mean volumes of the HT 29-1 tumours were plotted against the corresponding mean weights of the excised tumours. Each mean value of tumour volume and mass was obtained

FIG. 2.—Correlation between tumour volume (external caliper measurements) and tumour mass (excised tumour weight) in athymic mice (correlation coefficient 0·953).

from 3 animals which were killed between Days 7 and 56. There was a very good correlation of tumour volumes in the animals with tumours up to 800 mg. The regression line was calculated with a correlation coefficient of 0·953 between tumour mass and tumour volume. The mass of HT-29-1 tumours equivalent to 100 mm$^3$ of tumour volume was calculated as 148 mg.

*Mass/CEA and volume/CEA relationship*

The relationship between tumour mass and serum CEA was established in HT 29-1 and HT 29-2 tumour-bearing mice. In this series only tumours from animals without macroscopic evidence of tumour necrosis were evaluated. The mice were bled before tumour excision and were the same animals as those referred to in Fig. 2. The tumours were excised between Days 7 and 56 and the mean values of the tumour masses of 3 animals were correlated with the serum CEA concentration of the corresponding pooled sera. The correlation coefficient between tumour mass and circulating CEA was found to be 0·977 (n = 14). Individual tumour masses of animals with HT 29-2 tumours, when correlated with the individual serum CEA concentrations, also showed a correlation with a coefficient of 0·820 (n = 31).

Since there was an excellent relationship between tumour volume and tumour mass for the HT 29 sublines (Fig. 2) we determined a volume/CEA relationship instead of a tumour mass/CEA relationship to economize on animals. During the logarithmic growth phase, tumour volumes of individual mice could be determined 2–3 times together with a corresponding blood sample. The results presented in Fig. 3a, b also showed a linear relationship between circulating CEA and tumour volume. This finding proved that the release of CEA into the blood of the individual hosts by SLu-1 and SLu-2 sublines increased linearly with increasing

Fig. 3.—Correlation of tumour volume and serum CEA concentrations in individual SLu-1 (a) (correlation coefficient 0·839), and individual SLu-2 (b) tumour-bearing athymic mice (correlation coefficient 0·847).

tumour volumes during the logarithmic growth phase of the tumours. The correlation coefficient of tumour volume to CEA serum concentration was calculated to 0·839 for the SLu-1 (n = 55) and 0·847 for the SLu-2 subline (n = 23). The CEA blood concentration equivalent to 100 mm$^3$ tumour tissue was calculated to be 3·65 and 30·2 $\mu$g/l for the SLu-2 and the SLu-1 subline respectively.

### DISCUSSION

Our studies in nude mice produced evidence for an intrinsic relationship of *in vivo* CEA release and tumour growth of human colonic carcinoma HT 29 and SLu sublines releasing different amounts of CEA into the host circulation. In this animal model the serum concentration of the human tumour marker, CEA, proved to be an excellent growth parameter directly correlated with tumour size. A critical examination of the various phases of tumour growth indicated that this strict correlation was upheld only in the logarithmic growth phase. The shift to slower growth rates was linked to the dissociation of serum CEA and tumour volume leading to lower levels of circulating CEA. The critical tumour mass of s.c.-growing tumours at which the shift to slower growth rates occurred was 800–1200 mg independent of the tumour cell sublines. These tumours frequently showed macroscopic necrosis. The length of logarithmic growth phases of s.c. growing tumours depended highly on the doubling times of the cell line and required 6–8·5 doubling times to reach the shift in the growth rate. The CEA output of HT 29 and SLu sublines *in vivo* reflected the CEA release of the cells *in vitro*. The sublines releasing high amounts of CEA *in vitro* also retained this characteristic *in vivo*, though we have to expect CEA catabolism in the mouse (Thomas & Hems, 1975). The findings of Miwa *et al.* (1976) concerning the linear relation of tumour mass and circulating CEA have been confirmed and widely extended in our investigations. The conflicting results published by Stragand *et al.* (1980), who did not find a correlation between tumour size and serum CEA levels with the LoVo adenocarcinoma cell line transplanted into nude mice, may come in part from evaluating animals with tumours at different growth phases. In a recent publication by Lewis & Keep (1981) no clear correlation between serum CEA levels and tumour size was reported. They used xenografted tumour tissue which developed central necrosis as a consistent feature when grown in nude mice.

Necrosis as an interfering event for the linear relation of a serum tumour marker and tumour size has been described for AFP-secreting human teratomas in immunosuppressed mice (Raghavan *et al.*, 1980). Our observations that fluctuations of the serum CEA concentration occurred only after the shift from logarithmic to slower growth rates, associated with the frequent appearance of macroscopic necrosis, might also reflect a direct interference of tumour necrosis with CEA release. Experiments to characterize a possible influence of tumour necrosis on CEA release are in progress.

The authors wish to thank Ms S. Glock for excellent technical assistance.

### REFERENCES

Carrel, S., Sordat, B. & Merenda, C. (1976) Establishment of a cell line (Co-115) from a human colonic carcinoma transplanted into nude mice. *Cancer Res.*, **36**, 3978.

Committee on Standardized Nomenclature for Inbred Strains of Mice (1968) Standardized nomenclature for inbred strains of mice: Fourth listing. *Cancer Res.*, **28**, 391.

Committee on the Care and Use of the "Nude" Mouse (1976) Guide for the care and use of the nude (thymus deficient) mouse in biomedical research. *ILAR News*, **19**, M1-M20.

DiPersio, L., Dingle, S., Michael, J. G. & Pesce, A. J. (1980) Release of $\beta$2-microglobulin by human tumors grown in nude mice. *Expl. Cell. Biol.*, **48**, 429.

Gold, P. & Freedman, S. O. (1965) Specific carcinoembryonic antigens of human digestive system. *J. Exp. Med.*, **122**, 467.

Helson, L., Das, S. K. & Hajdu, S. I. (1975) Human neuroblastoma in nude mice. *Cancer Res.*, **35**, 2594.

Holyoke, E. D., Chu, T. M. & Murphy, G. P. (1975) CEA as a monitor of gastro-intestinal malignancy. *Cancer*, **35**, 830.

Lewis, J. C. M. & Keep, P. A. (1981) Relationship of serum CEA levels to tumour size and CEA content in nude mice bearing colonic tumour xenografts. *Br. J. Cancer*, **44**, 381.

Lo Gerfo, P. & Herter, F. (1975) Carcinoembryonic antigen and prognosis in patients with colon cancer. *Ann. Surg.*, **181**, 81.

Miwa, M., Sakura, H., Kawachi, T. & 5 others (1976) Serum carcinoembryonic antigen level and transplanted colonic tumor size in nude mice. In: *Oncodevelopmental Gene Expression* (Eds Fishman & Sell) New York; Academic Press p. 423.

Pantelouris, E. M. (1968) Absence of thymus in a mouse mutant. *Nature*, **217**, 370.

Papsidero, L. D., Kuriyama, M., Wang, M. C., Horoszewicz, J., Leong, S. S., Valenzuela, L., Murphy, G. P. & Chu, T. M. (1981) Prostate antigen: a marker for human prostate epithelial cells. *J. Natl Cancer Inst.*, **66**, 37.

Pesce, A. J., Bubel, H. C., DiPersio, L. & Michael, J. G. (1977) Human lactic dehydrogenase as a marker for human tumor cells grown in athymic mice. *Cancer Res.*, **37**, 1993.

Raghavan, D., Gibbs, J., Nogneira Costa, R., Kohn, J., Orr, A. H., Barrett, A. & Peckham, M. J. (1980) The interpretation of marker protein assays: a critical appraisal in clinical studies and a xenograft model. *Br. J. Cancer*, **41**, (Suppl. IV), 191.

Rittgers, R. A., Steele, G., Zamcheck, N. & 6 others (1978) Transient carcinoembryonic antigen (CEA) elevations following resection of colorectal cancer: A limitation in the use of serial CEA levels as an indicator for second-look surgery. *J. Natl Inst.*, **61**, 315.

Rygaard, J. & Povlsen, C. O. (1969) Heterotransplantation of a human malignant tumor to "nude" mice. *Acta Pathol. Microbiol. Scand.*, **77**, 758.

Sordat, B., Fritsche, R., Mach, J. P., Carrel, S., Ozello, L. & Cerottini, J. C. (1974) Morphological and functional evaluation of human solid tumors serially transplanted in nude mice. In *Proceedings of the First International Workshop on Nude Mice.* (Eds. Rygaard & Povlsen) Stuttgart: Gustav Fischer Verlag, p. 269.

Staab, H. J., Anderer, F. A., Ahlemann, L. M. & Frommhold, W. (1979) CEA monitoring and management of patients with carcinomas of the breast, lung, bladder, kidney and esophagus in radiotherapy. In *Carcinoembryonic Proteins, Vol II* (Ed. Lehmann), Amsterdam; Elsevier/North Holland Biomedical press, p. 151.

Stragand, J. J., Yang, L. Y. & Drewinko, B. (1980) Serum CEA levels in a human colonic adenocarcinoma (LoVo) xenograft system. *Cancer Letters*, **10**, 45.

Thomas, P. & Hems, D. A. (1975) The hepatic clearance of circulating native and asialo carcinoembryonic antigen by the rat. *Biochem. Biophys. Res. Com.*, **67**, 1205.